# Incremental Learning of Goal-Directed Actions in a Dynamic Environment by a Robot Using Active Inference

**DOI:** 10.3390/e25111506

**Published:** 2023-10-31

**Authors:** Takazumi Matsumoto, Wataru Ohata, Jun Tani

**Affiliations:** Cognitive Neurorobotics Research Unit, Okinawa Institute of Science and Technology, Okinawa 904-0495, Japan; takazumi.matsumoto@oist.jp (T.M.); wataru.ohata@oist.jp (W.O.)

**Keywords:** incremental learning, free energy principle, active inference, goal-directed action planning

## Abstract

This study investigated how a physical robot can adapt goal-directed actions in dynamically changing environments, in real-time, using an active inference-based approach with incremental learning from human tutoring examples. Using our active inference-based model, while good generalization can be achieved with appropriate parameters, when faced with sudden, large changes in the environment, a human may have to intervene to correct actions of the robot in order to reach the goal, as a caregiver might guide the hands of a child performing an unfamiliar task. In order for the robot to learn from the human tutor, we propose a new scheme to accomplish incremental learning from these proprioceptive–exteroceptive experiences combined with mental rehearsal of past experiences. Our experimental results demonstrate that using only a few tutoring examples, the robot using our model was able to significantly improve its performance on new tasks without catastrophic forgetting of previously learned tasks.

## 1. Introduction

Active inference (AIF) has steadily gained prominence as a theory of cognition that can be used as a framework to design artificial agents. Typically, brain-inspired models are based on the so-called forward model, which predicts the sensory state after an action is executed [1,2]. This can be extended to goal-directed behavior by providing a preferred (goal) sensory state as the distal (end) step, and then inferring the optimal sequence of actions under some constraint, such as minimum travel time. On the other hand, AIF agents, through application of the free-energy principle [3] and Bayes theorem, infer actions that minimize Bayesian surprise. This induces agents to act so as to remain in a limited set of preferred states [4], and has also been extended into goal-directed behavior by providing a preferred sensory state and finding an optimal action policy that minimizes expected free energy in the future by policy rollout [5,6].

Our previous work [7] expanded the aforementioned goal-directed AIF scheme by applying the concept of teleology, an idea that originated in ancient Greece with Plato and Aristotle. Under a teleological framework, phenomena appear not via their causes, but by their end results. That is, actions are always taken in order to achieve a goal or purpose; thus, actions can be explained by specifying the state of the environment or the event to which the actions are directed [8]. Although teleology has been dismissed as a scientific means of explaining physical phenomena, the idea of teleology can be used to model goal-directed action generation. Under such a framework, goal-directed actions can be characterized by three elements: (1) a goal, (2) actions to achieve that goal, and (3) physical constraints on actions [9]. It follows that when two of the these three elements are available, the final element can be inferred. Csibra et al. [9] showed that this inference apparently occurs in human infants, and our work also shows similar inference behavior in robot experiments.

On a similar bio-inspired basis, there is growing interest in employing incremental learning for artificial agents [10]. Incremental learning, also known as continual or lifelong learning, is regarded as an essential part of the developmental process of natural intelligence [11]. Traditionally, all training data necessary for learning a task are prepared before a training phase, after which testing with novel data can be conducted. In this case, a new network is trained if a new task is to be learned. While this may be sufficient for simple, static systems, in a dynamic environment with changing tasks, this approach rapidly becomes impractical. Additionally, as neural networks and datasets become larger, it is far more efficient to reuse an existing trained network and to extend its capabilities than to restart from scratch.

One phenomenon that all incremental learning techniques encounter is that of *catastrophic forgetting*, in which sequential learning of new concepts leads to a sudden loss of knowledge of previously learned concepts [12,13]. In general, during incremental learning, it is desirable to keep previously learned knowledge while adding new knowledge. While several approaches address this issue, our proposed scheme uses a form of mental rehearsal [13,14] using sequences generated by the trained neural network prior to incremental learning. This approach has been documented as directly impacting acquisition and retention of motor skills in humans [15], and has also been successfully applied to artificial agents using recurrent neural networks [16].

This study demonstrates how a physical humanoid robot generating goal-directed action plans can learn to dynamically adapt to changing situations by incremental learning from a small number of human-tutored examples. To achieve this, we leveraged our previously proposed model of goal-directed action planning based on active inference and teleology (T-GLean) [7]. This model applies the free-energy principle and uses the predictive coding-inspired variational recurrent neural network (PV-RNN) [17] to generate output in the continuous domain. A robot controller converts predicted proprioceptive states into robot joint angles and drives the robot joints appropriately. At the same time, sensor data containing exteroception of the object’s position and proprioception are fed back to the network. A human experimenter serves both to introduce interference into the robot task and also to tutor the robot in how to complete the task when the robot does not respond appropriately. The robot uses these tutored examples together with a sample of generated plans as a mental rehearsal and conducts additional training in an offline incremental learning phase. Details of our model and incremental learning scheme are given in Section 3.

Our proposed scheme is evaluated in Section 4 with a series of experiments demonstrating the construction of a functional incremental learning model using a humanoid robot. Experiment 1 utilized training data of the basic task, wherein the robot picks up an object placed within a workspace and places it at a selected goal position, and trains networks with different network parameters in order to establish a baseline from which to conduct incremental learning. Task performance is evaluated using the displacement of the final object position from the actual goal position when evaluated on untrained object-goal position combinations. In Experiment 2, we introduced deliberate interference while the robot is executing its plan, either by physically moving the object or changing the goal position. Since PV-RNN is able to adapt to certain changes in the environment at the start of plan generation, we examined how much adaptation is possible without additional learning. Finally, in Experiment 3, we provided the network with several human-tutored examples in order to accomplish the tasks in the case of interference. In this case, the human experimenter grasped the robot’s arms and physically guided its movements in order to pick up the object and move it to the goal. After two rounds of incremental learning, we evaluated not only its performance on new tasks, but also its performance on previously learned tasks to confirm that task performance on previously learned tasks had not degraded.

## 2. Related Studies

**Figure 1:**
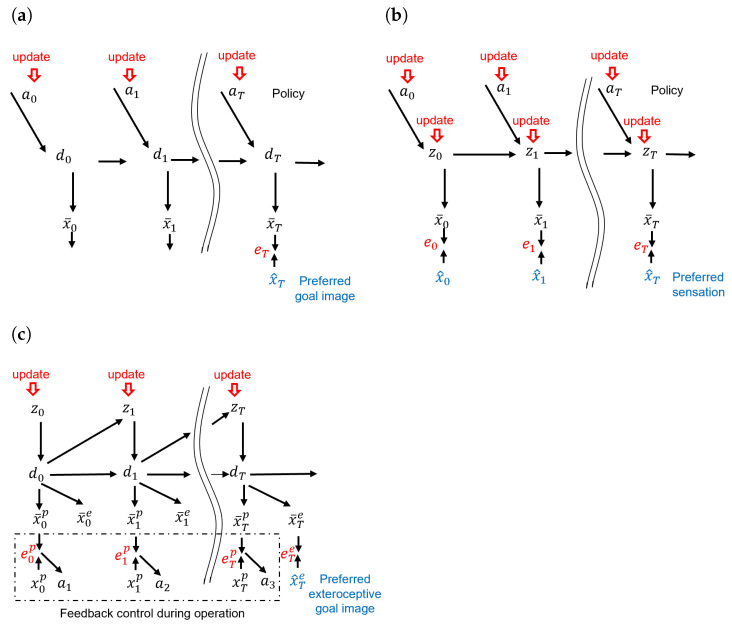
Models for generating goal-directed behaviors. (**a**) Forward model using latent variables, (**b**) active inference model using probabilistic latent variables, and (**c**) goal-directed latent variable inference (GLean). Here, errors between the sensory predictions and the observations are denoted by $e_{t}$.

Many brain-inspired models that generate goal-directed action plans have been proposed in the literature. Figure 1 illustrates three approaches, the forward model, the active inference model, and our previously proposed GLean model [7].

These approaches are based on the forward model predict sensory outcomes of each action [1,2,18]. Generally, an action plan (policy) consists of multiple future steps of proprioception, i.e., joint angles, and predicted observed states, such as the position of a robot in coordinate space. In a goal-directed problem, such as robot navigation, the agent has a goal image (sensory state) and will attempt to match its observations of the environment to that goal image by generating actions. The forward dynamics model and the kinematic model cascaded in time are depicted in Figure 1a. In this graphical representation, $a_{t}$, $d_{t}$, and ${\bar{x}}_{t}$ denote the action, deterministic latent variable, and sensory prediction at time step *t*, respectively. ${\hat{x}}_{T}$ represents the sensory goal image at the distal step *T*. After the network is trained, by minimizing the error between the predicted sensory state and goal image at *T*, an optimal sequence of actions can be inferred using the forward model, given a constraint such as minimal motor torque, as well as motor torque input at each time step. This allows the forward model to efficiently generate long-horizon plans, as opposed to traditional control approaches such as model predictive control (MPC) that become computationally intractable beyond short time horizons [19]. Murata et al. [20] demonstrated a scheme using long short-term memory (LSTM) networks and convolutional variational autoencoders to map goal images to visuomotor predictions to allow a robot to operate in collaboration with a human by observing the human changing the environment. We used a similar approach [21] and, in the current study, we expand the scenario such that the human experimenter can both interfere with the robot’s task as well as physically guide it in order to reach the goal.

Goal-directed planning to reach preferred sensory states can be formulated using the framework of active inference [22,23,24], based on the free-energy principle [3]. By implementing active inference using a deep or recurrent neural network, so-called deep active inference agents can exhibit goal-directed behavior by defining a preferred sensory state or latent state [25,26]. Active inference has previously been applied to robot motor control [27], action planning [28,29], and goal inference [30]. Figure 1b depicts an active inference-based approach incorporating a Bayesian perspective. In this representation, the probabilistic latent variables $z_{0:T}$ (with the notation 0:*T* representing a sequence from time step 0 to *T*) and an action policy $a_{0:T}$ can be inferred by minimizing the expected free energy. In order to find the optimal policy, a Markov chain Monte-Carlo (MCMC) sampling technique in the action space can be applied or, if searching in latent space with discrete actions, a more efficient Monte-Carlo Tree Search (MCTS) can be applied [29].

(1) $$G_{aif}=\sum_{t>t_{c}}^{T}-E_{q(z_{t},x_{t}|\pi )}\left[\;\;\;\underset{\mathrm{epistemic}\;\mathrm{value}}{\underset{︸}{\log p\left(z_{t}|x_{t}\right)-\log q\left(z_{t}|\pi \right)}}\;\;+\underset{\mathrm{extrinsic}\;\mathrm{value}}{\underset{︸}{\log p(x_{t})}}\right]$$

As shown in Equation (1), minimizing the expected free energy *G* maximizes the so-called epistemic and extrinsic values. The epistemic value represents the expected information gain with predicted outcomes or, in other words, the expected decrease in uncertainty with regard to the hidden state inferred from the sensory observation. The extrinsic value represents to what extent expected sensory outcomes in the generated plan are consistent with preferred outcomes. Here, π is an action policy, $p(z_{t}|x_{t})$ is the posterior distribution, $q(z_{t}|\pi )$ is the approximate posterior predictive distribution, $p(x_{t})$ is sensory evidence, and $t_{c}$ is the current time step. The resulting optimal policy can then be used in order to generate actions that move the agent [5,6,28]. For example, Dreamer [31] is a model-based reinforcement learning agent that can learn a world model and can generate action plans by “imagination” in the latent space, which is similar in concept to our planning approach. The development of our approach and its key features are as follows.

Matsumoto and Tani [21] proposed a variation of the active inference approach to goal-directed action plan generation, goal-directed latent variable inference (GLean), which was implemented using the PV-RNN network architecture employing the free-energy principle. Under this model, an optimal goal-directed plan is obtained by inferring the lower-dimensional probabilistic latent space, rather than higher dimensional action space. This approach has been implemented previously using a deterministic multi-layered RNN for hierarchical action planning [32], and is also analogous to sub-goal embedding in latent space using model-based reinforcement learning [33]. The graphical model of the scheme is depicted in Figure 1c, wherein the proprioception ${\bar{x}}_{t}^{p}$ and the exteroception ${\bar{x}}_{t}^{e}$ at each time step *t* are predicted by the learned generative model using the probabilistic latent variable $z_{t}$ and deterministic latent variable $d_{t}$. For a given preferred goal ${\hat{x}}_{T}$ represented by an exteroceptive state at the distal step *T*, a proprioceptive–exteroceptive sequence to reach this goal state is found by inferring an optimal sequence of the posterior predictive distribution of the latent variables $q(z_{1:T}|{\hat{x}}_{T})$ by means of minimizing the expected free energy $G_{glean}$ as shown in Equation (2).

(2) $$G_{glean}=\underset{\mathrm{goal}\;\mathrm{error}}{\underset{︸}{-E_{q(z_{T}|{\hat{x}}_{T})}\left[\log p(x_{T}|d_{T})\right]}}+\underset{\mathrm{complexity}}{\underset{︸}{\sum_{t=t_{c}}^{T}D_{KL}\left[q\left(z_{t}|{\hat{x}}_{T}\right)∥p\left(z_{t}|d_{t-1}\right)\right]}}$$

The GLean model was further developed in our previous study [7] into the T-GLean model, which learned a separate preferred goal state in continuous space for which a preferred goal can be given as a target, rather than by minimizing the error between an observation and a preferred sensory state, as in previous active inference-based approaches. This was demonstrated in our previous study, where our model not only generated a sequence of actions for a given goal, but also predicted what the goal should be based on the actions it observes. In a normal situation, the error between prediction and reality is small and the actions are sufficient to reach the goal, but as we explore in this study, in some cases this error rises, and this can have a significant impact on future predicted actions and goals.

Another aspect of active inference that has gained attention recently is demonstrating agents’ understanding and explaining their actions [34]. In our study, and others, such as [20], the agent is expected to implicitly understand what the human user wishes to accomplish from a combination of the provided goal and sensory inputs, and then to express this understanding as a series of actions, as well as by inferring the goal state. In a future study, we are considering a more explicit way for the agent to express its understanding of its environment, such as by using a language model.

Incremental learning, also known as continual learning, is part of a broad family of learning techniques including transfer learning and lifelong learning. Fundamentally, the goal of incremental learning is to efficiently gain new knowledge without losing old knowledge, the so-called catastrophic forgetting problem [12,13]. Incremental learning can be considered in three types of problem domains [35]: (1) task incremental, in which a task identifier is provided in both training and test time, (2) domain incremental, in which a task identifier is provided during training, but is not inferred at test time, and (3) class incremental, in which a task identifier must be inferred at test time. In this study, we focus on task-incremental learning, with the task being typical actions required for the robot to complete its objective of carrying an object to a goal position. In the field of machine learning, incremental learning has been extensively explored, both with traditional ML methods such as support vector machines [36] and with neural networks. Tani [16] applied incremental learning to robot navigation, using a “hippocampal database” in order to store generated output from the neural network to combine with new learning examples. This kind of mental simulation or rehearsal is considered vital in acquiring and retaining motor skills in humans [15] and effective for avoiding the catastrophic forgetting of knowledge during sequential learning [14]. In this study, we apply a mental rehearsal scheme combined with a small number of human tutoring examples to achieve incremental learning of new tasks.

Generally, incremental learning can be categorized into one of three approaches [37]: (1) replay, in which samples of previous training sequences are retained and reused, (2) regularization, which limits the flexibility of the neural network in learning new tasks, and (3) parameter isolation, which segments the neural network and prevents previously learned network weights from changing. A replay-based approach is the simplest, in which samples of training data used to train a network are retained for subsequent incremental learning, allowing the network to relearn past tasks. While simple, replay-based methods have a major disadvantage in that storing old training data is not always possible in the real-world, and can significantly increase the memory demands of training [38].

Regularization-based methods attempt to limit changes to the learned weights when learning new tasks, in order to preserve knowledge of previously learned tasks. Fine-tuning, where a trained neural network is re-trained on new data, but with a reduced learning rate to reduce the rate of change in the weights, could be considered the simplest realization of this approach. Other methods add a regularization term to the loss function that increases the cost of changing previously learned weights [39,40]. Perhaps the most well-known method within this family of methods is Learning without Forgetting (LwF) [41], which uses knowledge distillation loss [42] between the previous and incrementally trained model. An issue with this approach is that a balance must be empirically determined between retaining previous task knowledge and acquiring new knowledge. Finally, parameter isolation approaches typically learn an additional weight mask or sub-network per task [43,44,45]. While such approaches can be effective, they generally limit the number of tasks that can be learned, dividing network resources in a predefined manner or requiring additional parameters per new task. Under this categorization, our proposed approach is closest to LwF and similar regularization methods, leveraging the architecture of PV-RNN for regularization and using mental simulation to avoid the disadvantages of replay-based approaches.

## 3. Methodology

In this section, we first review our T-GLean model for goal-directed plan generation and the PV-RNN architecture it is built upon, focusing on the most pertinent parts of the model. Following this, we describe our approach to incremental learning, with particular attention to how catastrophic forgetting and learning efficiency are managed. Details of how we conducted incremental learning in this study are presented in the following Section 4.

### 3.1. Model Overview

As noted previously, this study builds upon our proposed T-GLean model [7], which was an extension of our previous GLean model [21] with a teleology-inspired goal representation. While this study focuses on the incremental learning framework, the differences in the model architecture employed in this study compared to the model described in [7] are summarized as follows:

1. Output layer connections: in our previous study a single output layer concatenated all output dimensions, but in this study we employ separate output layers for more flexibility;
2. Output layer type: our previous studies employed a variation of softmax output, but in this study we employ a Gaussian output model for proprioception, exteroception and goals, with associated changes in the free energy computation as described in Equation (12);
3. Hyperparameter settings: in addition to adjusting hyperparameters for the task undertaken, we can now adjust the meta-prior value independently for past and future windows, as shown in Experiment 2.

Our model utilizes the PV-RNN architecture, which leverages the idea that multiple timescale RNNs [46] can enable development of a functional hierarchy from training data.

**Figure 2:**
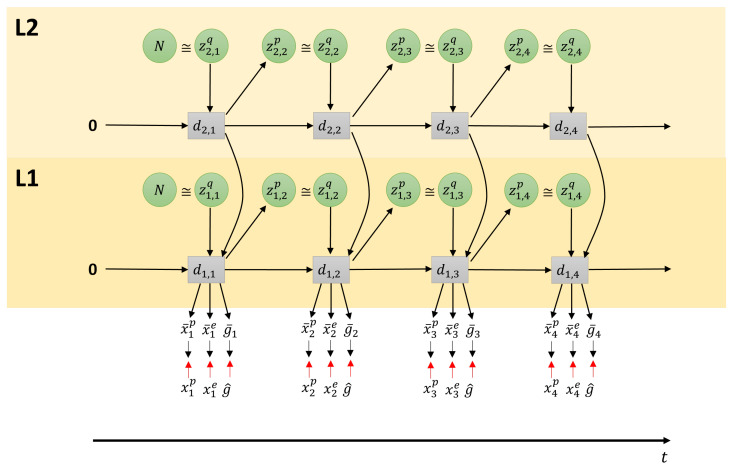
Graphical description of the network architecture used in this study. The highlighted regions, labelled L1 and L2, represent the two layers of the network.

In this study, the objective of the robot is to move the red colored object in the workspace to the goal position. Our model operates in continuous space, generating four outputs at each time step: proprioception, exteroception, goal position, and distal step probability. In our current model, these are separate output layers, and it cannot be assumed that the outputs are correlated. A graphical representation of this model is shown in Figure 2, with a two layer PV-RNN unrolled in time. The preferred goal position $\hat{g}$ is given as a scalar value representing the coordinate of the goal position. Actions that the robot should execute in order to move the object to the goal depend not only on the positions of the object and goal, but also on the state of the robot itself. In addition, as can happen in the real world, in our experiments, the task can change while the robot is executing its plan.

By giving a preferred goal at each time step, the posterior predictive distribution *q* can be updated in order to minimize the divergence between the preferred goal and the predicted goal at each time step. This update of the posterior predictive distribution at each time step is accomplished using backpropagation through time (BPTT) [47]. By sampling the posterior predictive distribution through the deterministic latent variable, the predicted proprioceptive and exteroceptive trajectory leading to the goal is generated. This proprioceptive–exteroceptive trajectory is the goal-directed plan which is executed one step at a time by the robot.

This scheme is different from a conventional active inference-based goal-directed planning scheme, such as in [6], in which the expected free energy is computed for every possible policy by means of policy rollout. The optimal policy, which has the lowest expected free energy, can then be selected to generate actions to reach the goal. In contrast, in our proposed model, optimization to minimize the expected free energy is conducted in the space of the posterior predictive distribution. Once the optimal approximate posterior distribution at each time step is determined, the corresponding proprioception and exteroception at each step are determined by mapping through the deterministic latent variable. Under the AIF framework, the robot controller takes the predicted proprioception as an action policy, converting it into optimal motor control sequences and commanding the robot to move appropriately.

Each layer, indexed by *l*, contains the probabilistic latent variable *z* and deterministic latent variable *d*. At each time step *t*, $z_{l,t}^{p}$ is sampled from the prior distribution, and $z_{l,t}^{q}$ from the approximate posterior distribution for the past and the posterior predictive distribution for the future, as well as the deterministic latent variable $d_{l,t}$. The output of the bottom layer is connected to four output layers: proprioception ${\bar{x}}_{t}^{p}$, exteroception ${\bar{x}}_{t}^{e}$, expected goal ${\bar{g}}_{t}$, and distal step probability ${\bar{s}}_{t}$.

The preferred goal ${\hat{g}}_{t}$ is given at all time steps as a target, and can change if the goal changes. The observed proprioception $x_{t}^{p}$ and exteroception $x_{t}^{e}$ are computed from robot sensor data and used as targets for error minimization. Another key development from our previous implementation of this model is in computation of the output. The predicted output of proprioception, exteroception, and goal are computed as a Gaussian distribution. In practice, the robot controller only uses the mean value; however, by modeling typical fluctuations experienced by physical motors and sensors in the output layer, only large errors caused by deliberate deviations should backpropagate to the PV-RNN layers. We note that due to this change, network parameters and free energy values shown in this current study cannot be compared to those in our previous studies. We discuss the Gaussian output scheme further in the following subsection.

Finally, the distal step probability $s_{t}$ is a softmax encoding with two dimensions representing the probability of the robot having reached the goal. During training, these are known and given as [0.0,1.0] before reaching the goal and [1.0,0.0] after reaching it. For details on the softmax computation, please refer to [7]. Note that in order to avoid overcrowding the figures, $s_{t}$ is omitted from graphical representations of the network.

### 3.2. Learning

The forward computation of PV-RNN is given in Equation (3), which is based on the formula of the continuous-time recurrent neural network (CTRNN) [48,49]. Multiple-timescale RNN (MTRNN) [46] uses multiple layers of CTRNN with different time constants assigned to each layer, building a hierarchical structure. Generally, we assume that higher layers of the network have longer time constants.

(3) $$\begin{array}{cc} h_{l,t} & =\left(1-\frac{1}{\tau^{l}}\right)h_{l,t-1}+\frac{1}{\tau^{l}}\left(W_{d,d}^{l,l}d_{l,t-1}+W_{z,d}^{l,l}z_{l,t}+W_{d,d}^{l+1,l}d_{l+1,t}\right),\\ d_{l,t} & =\tan h(h_{l,t}),\\ d_{l,0} & =0. \end{array}$$

$\tau^{l}$ is the time constant of layer *l*. The internal state of each RNN unit $h_{l,t}$ at time step *t* and level *l* is computed as a sum of the connectivity weight multiplication of $z_{l,t}$, $h_{l,t-1}$, and $h_{l+1,t}$ (if *l* is not the top layer). The connectivity weight matrix W is indexed from layer to layer and from unit to unit. For brevity, bias terms have been omitted.

PV-RNN further builds on MTRNN with probabilistic latent variables *z*, which follow Gaussian distributions. Each sample of the prior distribution $z_{t}^{p}$ for layer *l* is computed as shown in Equation (4). For brevity, a single sequence is assumed.

(4) $$\begin{array}{cc} \mu_{l,t}^{p} & =\left\{\begin{array}{cc} 0, & \mathrm{if}\;t=1\\ \tan h(W_{d,z^{\mu^{p}}}^{l,l}d_{l,t-1}), & \mathrm{otherwise} \end{array}\right.\\ \sigma_{l,t}^{p} & =\left\{\begin{array}{cc} 1, & \mathrm{if}\;t=1\\ \exp(W_{d,z^{\sigma^{p}}}^{l,l}d_{l,t-1}), & \mathrm{otherwise} \end{array}\right.\\ z_{l,t}^{p} & =\mu_{l,t}^{p}+\sigma_{l,t}^{p}⊙\epsilon . \end{array}$$

ϵ is a random noise sample such that ϵ∼N(0,I). ⊙ represents the element-wise product operation.

Similarly, each sample of the approximate posterior distribution $z_{t}^{q}$ is computed as shown in Equation (5), where A is a learned variable that is used to compute the mean and standard deviation of $z_{t}^{q}$ at each step in a sequence.

(5) $$\begin{array}{cc} \mu_{l,t}^{q} & =\tan h(A_{l,t}^{\mu }),\\ \sigma_{l,t}^{q} & =\exp(A_{l,t}^{\sigma }),\\ z_{l,t}^{q} & =\mu_{l,t}^{q}+\sigma_{l,t}^{q}⊙\epsilon . \end{array}$$

To compute the output at each time step *t*, we assume that proprioception, exteroception, and goal follow a Gaussian distribution, in which the mean $\mu_{t}^{o}$ and standard deviation $\sigma_{t}^{o}$ are mapped from the bottom layer of the network, as in Equation (6).

(6) $$\begin{array}{cc} \mu_{t}^{o} & =\tan h(u_{t}^{o}),\\ \sigma_{t}^{o} & =\exp(s_{t}^{o}),\\ u_{t}^{o} & =W_{\mu }^{o}d_{1,t},\\ s_{t}^{o} & =W_{\sigma }^{o}d_{1,t}. \end{array}$$

Since this network minimizes free energy as its loss function, we will review the free energy formulation of our model. This is a modification of our previous formulation [7], with the previously mentioned Gaussian output layer. In the following explanation, for simplicity, we assume there is a single layer PV-RNN only.

During learning, the evidence free energy shown in Equation (7) is minimized by iteratively updating the approximate posterior of *z*, as well as the learned parameters *W* at each time step for all training sequences. The delta error between the generated output and the training output target, along with the Kullback–Leibler (KL) divergence between the approximate posterior and prior, is backpropagated from the end of the training sequence to the first time step. The parameters of the network are updated in the direction of minimizing this delta error using the Adam optimizer [50].

(7) $$\begin{array}{cc} F(x,\hat{g},\hat{s},z)= & \sum_{t=1}^{T}(w\cdot \underset{\mathrm{complexity}}{\underset{︸}{D_{KL}\left[q\left(z_{t}|x_{t:T},{\hat{g}}_{t:T},{\hat{s}}_{t:T}\right)∥p\left(z_{t}|d_{t-1}\right)\right]}}\\ \; & -\underset{\mathrm{accuracy}}{\underset{︸}{E_{q(z_{t}|x_{t:T},{\hat{g}}_{t:T},{\hat{s}}_{t:T})}[\log p\left(x_{t},{\hat{g}}_{t},{\hat{s}}_{t}|d_{t}\right)]}}). \end{array}$$

x, $\hat{g}$, and $\hat{s}$ are the observed sensory states, preferred goal, and distal step probability, respectively. Free energy is modified by inclusion of the meta-prior *w*, which weights the complexity term. *w* is a hyperparameter that affects the degree of regularization, similar to β in variational autoencoders [51]. Since we are dealing with sequences of actions, the free energy is a summation over all time steps in the sequence.

The first term, complexity, is computed as the KL divergence between the approximate posterior and prior distributions. This can be expressed as follows:

(8) $$D_{KL}\left[q\left(z_{t}|x_{t:T},{\hat{g}}_{t:T},{\hat{s}}_{t:T}\right)∥p\left(z_{t}|d_{t-1}\right)\right]=\int_{-\infty }^{\infty }q\left(z_{t}|x_{t:T},{\hat{g}}_{t:T},{\hat{s}}_{t:T}\right)\log\frac{q(z_{t}|x_{t:T},{\hat{g}}_{t:T},{\hat{s}}_{t:T})}{p(z_{t}|d_{t-1})}dz_{t}$$

Given μ and σ for both prior *p* and posterior *q* distributions from Equations (4) and (5), respectively, $p(z_{t}|d_{t-1})$ and $q(z_{t}|x_{t:T},{\hat{g}}_{t:T},{\hat{s}}_{t:T})$ can be expressed as follows:

(9) $$\begin{array}{cc} p(z_{t}|d_{t-1}) & =\frac{1}{\sqrt{2\pi {\left(\sigma_{t}^{p}\right)}^{2}}}\exp\left[-\frac{1}{2}{\left(\frac{z_{t}-\mu_{t}^{p}}{\sigma_{t}^{p}}\right)}^{2}\right],\\ q(z_{t}|x_{t:T},{\hat{g}}_{t:T},{\hat{s}}_{t:T}) & =\frac{1}{\sqrt{2\pi {\left(\sigma_{t}^{q}\right)}^{2}}}\exp\left[-\frac{1}{2}{\left(\frac{z_{t}-\mu_{t}^{q}}{\sigma_{t}^{q}}\right)}^{2}\right]. \end{array}$$

Thus, continuing from Equation (8), complexity can be analytically computed as:

(10) $$D_{KL}\left[\log\frac{\sigma_{t}^{p}}{\sigma_{t}^{q}}+\frac{{\left(\mu_{t}^{q}-\mu_{t}^{p}\right)}^{2}+{\left(\sigma_{t}^{q}\right)}^{2}}{2{\left(\sigma_{t}^{p}\right)}^{2}}-\frac{1}{2}\right].$$

For brevity, a single *z*-unit consisting of a (μ,σ) pair is assumed.

To compute the second term, accuracy, we first consider the log-likelihood for the network output as follows:

(11) $$\log p\left(x_{t}|d_{t}\right)=\log\frac{1}{\sqrt{2\pi }\sigma_{t}^{o}}\exp\left\{-\frac{1}{2}{\left(\frac{x_{t}-\mu_{t}^{o}}{\sigma_{t}^{o}}\right)}^{2}\right\}\propto -\frac{1}{2}{\left(\frac{x_{t}-\mu_{t}^{o}}{\sigma_{t}^{o}}\right)}^{2}-\log\sigma_{t}^{o}.$$

$\left(\mu^{o},\sigma^{o}\right)$ represents the mean and standard deviation of the output Gaussian, and $\bar{x}$ is a sample such that ${\bar{x}}_{t}∼N\left({\bar{x}}_{t}|d_{t};\mu_{t}^{o},\sigma_{t}^{o}\right)$. Again, for brevity, only a single output $\bar{x}$ is considered here.

The accuracy term in the lower bound can be expressed as follows:

(12) $$\begin{array}{c} E_{q(z_{t}|x_{t:T})}\left[\log P\left({\bar{x}}_{t}|d_{t}\right)\right]\propto E_{q(z_{t}|x_{t:T})}\left[-\frac{1}{2}{\left(\frac{{\bar{x}}_{t}-\mu_{t}^{o}}{\sigma_{t}^{o}}\right)}^{2}-\log\sigma_{t}^{o}\right]. \end{array}$$

Finally, maximizing the term in Equation (12) corresponds to minimizing the following, where the log-likelihood inside the expectation is analytically computed as follows:

(13) $$\frac{1}{2}{\left(\frac{{\bar{x}}_{t}-\mu_{t}^{o}}{\sigma_{t}^{o}}\right)}^{2}+\log\sigma_{t}^{o}.$$

In Section 4, for simpler visualization, we opt to remap the output of Equation (13) from [−∞,∞] to [0,∞] using the softplus function f(x)=log(1+exp(x)).

### 3.3. Plan Generation

An important feature of the T-GLean model and a key difference between it and conventional AIF-based schemes is that the expected goal state ${\bar{g}}_{t}$ is generated at every time step, and is independent of the sensory state at the distal step. Intuitively, this means that at every time step, the agent predicts the goal state that the on-going action sequence will eventually achieve. This not only allows the network to infer a goal state, but is also flexible in allowing the goal state to change continuously and does not require a fixed distal step.

In T-GLean, generation and backpropagation are performed in a *planning window* of length win. In the planning window, there is a *past window* of length $win^{p}$ and a *future window* of length $win^{f}$. In the past window, *evidence free energy* is minimized by updating the approximate posterior at each time step such that the latent variables result in an output close to the observed sensory sequence. In the future window, the error between the preferred goal and the expected goal output is minimized for all steps in the future window by optimizing the approximate posterior predictive distribution iteratively, which is accomplished by minimizing the *expected free energy*. This scheme is referred to as online error regression [52], and is analogous to methods described in [6,53]. This occurs in an online manner, with the robot controller executing each step of the current predicted proprioception at regular time steps. To avoid confusion between time steps in context of the network and the robot, we refer to time steps experienced by the robot as sensorimotor time steps.

**Figure 3:**
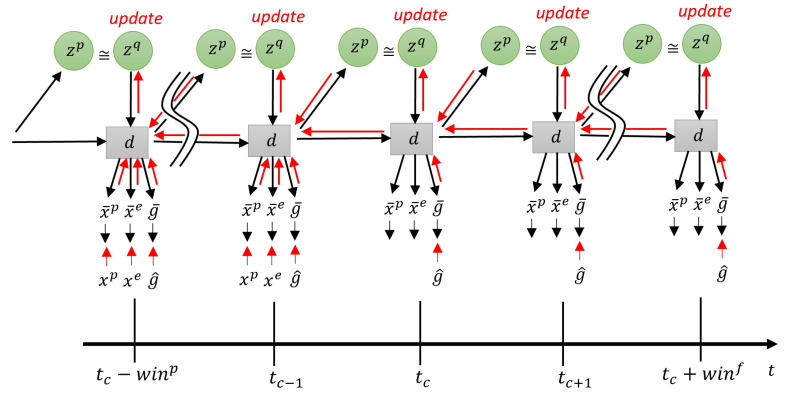
The network during planning with a planning window spanning from $t_{c}-win^{p}$ to $t_{c}+win^{f}$. Red lines indicate error backpropagation.

In this current implementation, the length of the planning window is fixed, while the past window starts at length 1 and is allowed to grow to half the length of the planning window. The current time step $t_{c}$ is one step ahead of the end of the past window. At the next sensorimotor time step, sensory information at $t_{c}$ becomes part of the past window, and $t_{c}$ moves forward one step, shrinking the future window. Once the past window is filled, the entire planning window slides one step to the right, discarding the oldest entry in the past window. This scheme is shown graphically in Figure 3.

During plan generation, the network minimizes the plan free energy $F_{\mathrm{plan}}$ by optimizing the approximate posterior distribution in the past window and posterior predictive distribution in the future window. The goal error is backpropagated from the end of the planning window at $t_{c}+win^{f}$, while the sensory error is backpropagated from $t_{c}-1$ until the beginning of the planning window at $t_{c}-win^{p}$. This delta error is propagated to $z^{q}$, which is then updated in the direction of minimizing the delta error and the KL divergence between approximate posterior and prior. Forward computation and error regression are repeated for a fixed number of iterations, such that an updated plan can be generated in a timely manner. In this study, we target a 10-Hz update rate; thus, forward and backward computation should be completed within approximately 100 ms.

The plan free energy $F_{\mathrm{plan}}$, as shown in Equation (14), consists of the sum of the evidence free energy $F_{e}$ in the past window and the expected free energy *G* in the future window.

(14) $$\begin{array}{cc} F_{e}\left(x,\hat{g},z\right) & =\sum_{t=t_{c}-win^{p}}^{t=t_{c}}(w_{p}\cdot D_{KL}\left[q\left(z_{t}|x_{t:t_{c}},{\hat{g}}_{t:t_{c}}\right)∥p\left(z_{t}|d_{t-1}\right)\right]\\ \; & -E_{q(z_{t}|x_{t:t_{c}},{\hat{g}}_{t:t_{c}})}\left[\log p(x_{t},{\hat{g}}_{t}|d_{t})\right]),\\ G(\hat{g},z) & =\sum_{t=t_{c}}^{t=t_{c}+win^{f}}(w_{f}\cdot D_{KL}\left[q\left(z_{t}|{\hat{g}}_{t:t_{c}+win^{f}}\right)∥p\left(z_{t}|d_{t-1}\right)\right]\\ \; & -E_{q(z_{t}|{\hat{g}}_{t:t_{c}+win^{f}})}\left[\log p({\hat{g}}_{t}|d_{t})\right]),\\ F_{\mathrm{plan}} & =F_{e}+G. \end{array}$$

The meta-prior *w* is a hyperparameter that adjusts the degree of regulation for the complexity term, i.e., the divergence between the approximate posterior and prior. The expected free energy *G* shown in Equation (1) and our formulation of expected free energy in Equation (14) differ significantly. This is because the conventional AIF-based approach in Equation (1) is calculated for a given policy, whereas there is no policy search in our approach since the proprioceptive sequence is determined from the optimized posterior predictive distribution, as described previously.

### 3.4. Incremental Learning

In order to implement incremental learning with our model, we consider two key factors mentioned previously, that is, how to learn new tasks efficiently, and how to avoid the problem of catastrophic forgetting. Before closing this current section, we describe our approach to incremental learning.

The approach we take in this current study follows from earlier work by Tani and Yamamoto [54], as well as some aspects from the Learning without Forgetting method [41]. After completing the initial training, as described in the previous section, the robot is tested with various untrained conditions. While it is expected that the robot can complete the trained task, what happens when the robot is presented with a sudden change in its situation? As can be deduced from Equation (14), in such a situation, the free energy of the system increases suddenly. The manner of the increase in free energy depends on how the robot’s situation has changed. If the sensorimotor observations no longer align with the expected exteroception image (such as when the object moves in a way that is unexpected) as inferred from past evidence and trained prior probability, then the evidence free energy $F_{e}$ rises. If the preferred goal to be reached is suddenly changed, while the robot is still executing actions that will result in reaching the previous goal, then the expected free energy *G* rises. In either case, as the robot is moving, the network has limited opportunity to optimize the approximate posterior distribution to minimize the free energy. By design of PV-RNN, the meta-prior *w* determines how far the approximate posterior can deviate from the trained prior, and it is possible that a solution that reduces free energy overall by either allowing the KL-divergence between the approximate posterior and trained prior to rise, or by ignoring the deviation between observations and the expected proprioceptive–exteroceptive or goal states. In our following experiments, we first isolate the effects of this behavior before determining the impact of incremental learning.

In this study, the robot is operating in real-time with a human experimenter, whose role is not only to set up tasks for the robot, but also to intervene in case the robot deviates from what the experimenter judges to be expected behavior. For example, if the object the robot is moving to grasp is moved, but the robot does not respond adequately, the robot may fail to grasp the object correctly. In this case, the human experimenter physically guides the robot’s arms toward the new object position, ensuring that it is grasped, and then allows the robot to continue to move to the goal. The robot can then save this new proprioceptive–exteroceptive sequence for incremental learning, which we refer to as a tutoring example. This is analogous to a caregiver guiding a child’s hands, with multi-modal information facilitating kinaesthetic learning of proprioceptive–exteroceptive states.

**Figure 4:**
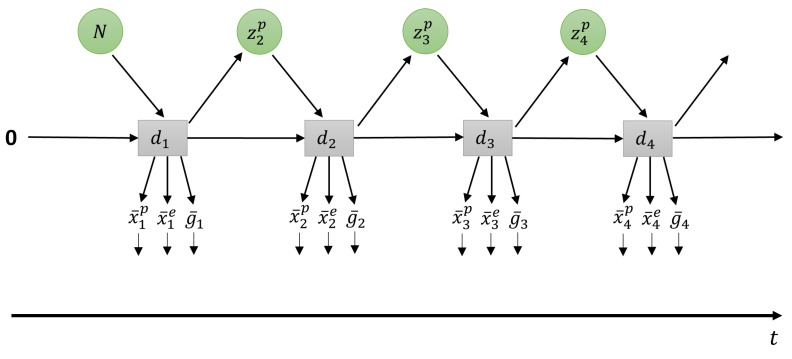
Generation using the prior distribution. Note that no backpropagation occurs in this case.

The author of the current study was the sole human experimenter, as required for development and testing in the following experiments. For efficiency, and since human supervision may be limited in real-world conditions, in this study, only a small number of such tutoring examples are created for each new task in which the robot shows reduced task performance. This approach is analogous to few-shot learning, which has also been applied in teaching robots by imitation [55]. These new tutoring examples are combined with rehearsal data to build a dataset for incremental learning. Whereas in experimental conditions, rehearsal data could be sampled from previous training data, this may be unrealistic in the real world. Instead, we generate rehearsal data using the prior distribution mapped through the deterministic latent variable as in Equation (4). This is also shown graphically in Figure 4.

Additionally, in this study, it is assumed that learning phases are distinct, that is, split between initial learning, testing, and incremental learning. During initial learning, the network learns a task (which we refer to as the basic task) from the full training dataset. Thereafter, in the test phase, the network is exposed to the basic task, as well as a new task, A. Human tutoring should result in recording of new tutoring examples when task A is introduced. During the incremental learning phase, the network combines tutoring examples with generated rehearsal data and begins the optimization process as in initial training. At the start of this phase, the previously learned approximate posterior distribution is reset, while the remaining learned parameters are left intact. Once incremental learning is complete, testing can be conducted again, repeating the incremental learning of another new task. The balance of new tutoring sequences and mental rehearsal sequences are important factors regulating how a few tutoring sequences can be used to acquire new tasks while mitigating catastrophic forgetting. To control the so-called concept drift [56], it is assumed that the task space expands gradually. That is, each new task is related to the previous task, such that the old network can still attempt to complete the new task. Under this scheme, more variation from the basic task can be learned over several incremental learning sessions, such as action sequences that are significantly longer than previously learned sequences.

## 4. Experiments

In order to demonstrate our proposed model, we conducted three experiments exploring different aspects of the model and incremental learning scheme. All three experiments were conducted using a humanoid robot, Torobo, a research humanoid robot manufactured by Tokyo Robotics Inc. with 16 degrees of freedom, torque-sensing back-drivable motors, and a stereo camera. The robot is connected to a controller that manages the torque and current levels of each motor. The controller receives joint angles at approximately 100 ms intervals from the predicted proprioception output of our model, which is implemented using LibPvrnn, a custom C++ library that implements PV-RNN. The source code for a development version of LibPvrnn that was used in this study is provided, together with the training data.

**Figure 5:**
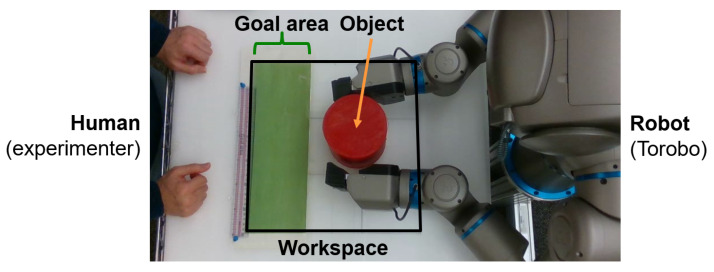
The experimental setup used in this study.

The robot was set up facing a platform with a workspace of approximately 36 cm × 36 cm, with the goal area defined as the top 12 cm of the workspace (see Figure 5). In each experiment, the objective of the robot is to pick up a red object approximately 11 cm in diameter from an arbitrary position in the workspace, and to place it at a specified goal position $\hat{g}$. The robot starts at a home position, before moving to grasp the object with both end effectors, lift it up, move it to the goal position, then set it down on the surface.

In this study, in order to simplify processing, full pixel data from the cameras are not used. Rather, an object tracking program takes images from the head-mounted camera and calculates two neck joint angles such that head of the robot always follows the object. From the position of the head, the object’s current position ((x,y) coordinates) in the workspace can then be calculated, and this coordinate is used as exteroception. The goal coordinate $\hat{g}$ is also given as a scalar value [0,1] corresponding to the position of the goal along the width of the goal area.

We note that due to application of object tracking in order to reduce computational workload, except at certain points such as when the robot grasps the object on the workspace surface, our experimental setup can be described as a fully observable Markov Decision Process (MDP). However, as our model employs an RNN architecture that can learn hidden environment states from past observations, we believe our scheme can be applied to more complex partially observable MDP (POMDP) problems as well.

In Experiment 1, we first conducted initial training of the basic task, which consists of picking up the object and placing it at the goal position. This training was performed on several networks with several different meta-prior settings. In order to quantify the robot’s performance on the task, we measured the *goal displacement*, that is, the distance between the preferred goal set by the experimenter and the final position of the object after the robot moves it. This measurement was performed programmatically using a camera mounted above the workspace that independently tracks the object, and compares it to $\hat{g}$ mapped to the workspace.

In Experiment 2, we introduced two new conditions that suddenly change the task while the robot was already executing a plan. In the first case, the object was moved by the experimenter just before the robot grasped it (referred to as task A), and in the second case, the goal was changed after the object was placed at the original goal (referred to as task B). In this experiment, we altered the meta-prior at test time, in order to evaluate how much task performance is affected by adjusting the regulation strength of the adaptation parameters alone.

Finally, in Experiment 3, we conducted incremental learning of task A followed by task B using tutoring examples, followed by testing of both new task acquisition and catastrophic forgetting of previous tasks. We also quantified the amount of human intervention that was required for the robot to complete the new task before and after incremental learning by measuring the additional force exerted on the robot’s joints by the experimenter.

In these experiments, we trained multiple networks using parameters shown in Table 1. Parameters that are changed in each experiment are noted in the respective subsections.

Before commencing each trial, the experimenter places the test object at a predefined position on the workspace, and sets the preferred goal position $\hat{g}$. Then, the error regression process is started. The tested object–goal position combinations should not be in the training or tutoring samples. The robot moves while the plan continually updates, until the distal step probability at the current time step $s_{t}$ exceeds a threshold, or a time limit is reached, at which point the robot stops moving and the trial ends. The experimenter can also override the distal step and either continue the trial (such as when tutoring the robot) or terminate the trial (such as when the robot comes into contact with the platform). This is repeated multiple times, with different untrained positions and goals, until the experiment is concluded.

### 4.1. Experiment 1: Initial Learning

In this experiment, we first established baseline performance from which we can compare the performance of later experiments. A well-trained network can generalize unlearned parameters, such as unlearned object positions, from a limited number of training samples, while also showing stable motion through habituated regions of each trajectory. For the basic task, we prepared 120 training samples in which the robot picked up the object and placed it at the specified goal position. These training samples were generated programmatically and then executed on the robot to record joint angle trajectories.

From our preliminary study, we determined that the meta-prior setting, particularly of the bottom layer, is important in generating network output. We theorize that since the bottom layer is situated between the output and the top layer, not only does its learned distribution directly impact the output, it also regulates how much error flows up to the top layer during backpropagation.

Thus, in order to evaluate the impact of the meta-prior setting, we prepared three configurations, with the top layer meta-prior fixed at $w^{l=2}=1.0$ and the bottom layer meta-prior set as $w^{l=1}=0.1$, 1.0, and 10.0, respectively. We represent this as a ratio between top and bottom meta-priors; for example, 1:10 represents a network with $w^{l=2}=1.0$ and $w^{l=1}=10.0$. From Equation (7), it can be hypothesized that smaller values of *w* may lead to improved generalization at the cost of increased noise, whereas larger values of *w* may generate more stable, habituated patterns at the cost of reduced generalization. In this experiment, *w* values used in training are also carried over to plan generation, with past $w_{p}$ and future $w_{f}$ meta-prior values also set to the same *w* value. It follows that the meta-prior may have a similar effect as in training, with larger *w* values leading to an increased chance of following the trained prior over adapting the approximate posterior to sensorimotor observations, and vice versa for smaller *w* values.

In order to efficiently collect statistics with a stochastic network architecture in a fair manner, we employed the following procedure:

1. Train 10 randomly initialized networks for each parameter set;
2. Sort the resulting networks by the loss at the end of training;
3. Discard the top three and bottom three networks, leaving four networks to test.

This procedure allowed us to train many networks with different random seeds, while rejecting any significantly overfitted or underfitted networks. It was important to undertake this filtering, as poorly trained networks can cause the robot to move in unpredictable and unsafe ways. At the same time, we avoid cherry-picking trained networks by test performance, as only the loss after training was considered. Each network was trained for 100,000 epochs, with the Adam optimizer parameters α=0.001, $\beta_{1}=0.9$, $\beta_{2}=0.999$. After the aforementioned procedure, we tested 12 trained networks with five test cases each, for a total of 60 trials. During online plan generation, the number of error regression iterations per robot sensorimotor step was set to 100 and α=0.1. The planning window length was 360 time steps, with the past window length starting at 1 and increasing until 180 time steps.

**Figure 6:**
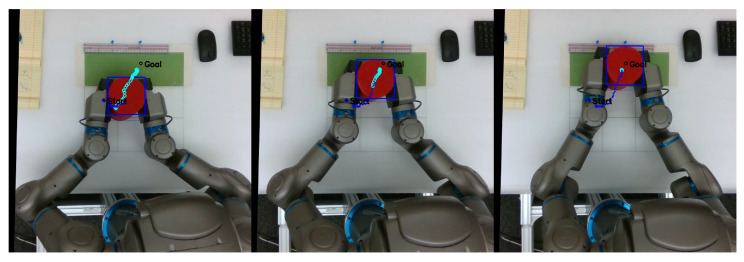
A sequence of three images showing an example of the robot completing the task. Each frame is overlaid with the past object positions in blue and the network predicted exteroception in cyan. The initial position and goal position are also labeled.

As mentioned previously, in order to evaluate the task performance of each network, we measured the distance between the center of the object and $\hat{g}$ as goal displacement, with lower displacement corresponding to better task performance. Figure 6 shows a sequence of frames from a video recorded from the overhead camera, overlaid with object position history, predicted exteroception ${\bar{x}}^{e}$, and goal position $\hat{g}$. A video with examples recorded from this series of experiments is available at https://youtu.be/lk-u6kuOfL8 (accessed on 3 September 2023).

**Figure 7:**
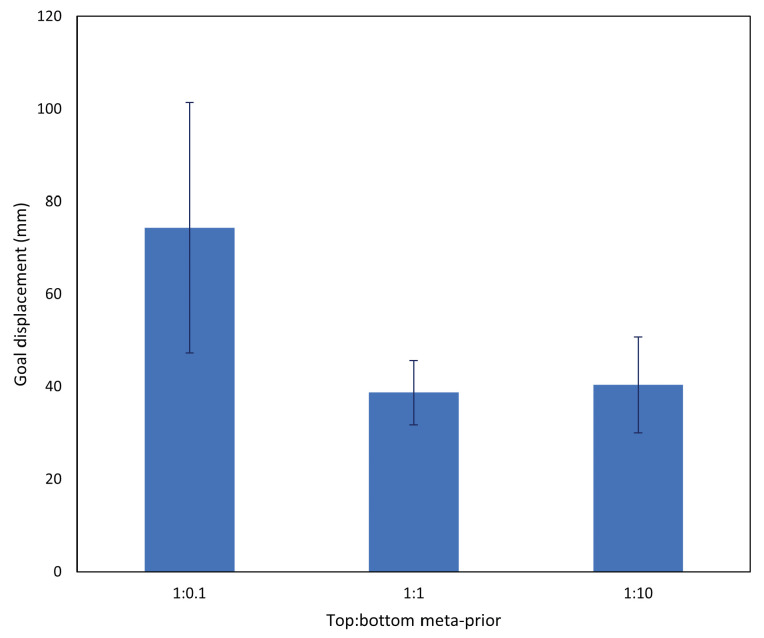
Goal displacement for the three tested meta-prior ratios. Each result is an average over 20 trials, with the standard deviation indicated by the error bars.

Each network was tested with five untrained test cases, with results collated and averaged in Figure 7. The networks with *w* ratio 1:0.1 (i.e., bottom layer $w^{l=1}=0.1$) performed significantly more poorly than the other two configurations. This was due to an inability of the 1:0.1 network to generate a stable future trajectory, indicating an inadequately trained prior distribution.

**Figure 8:**
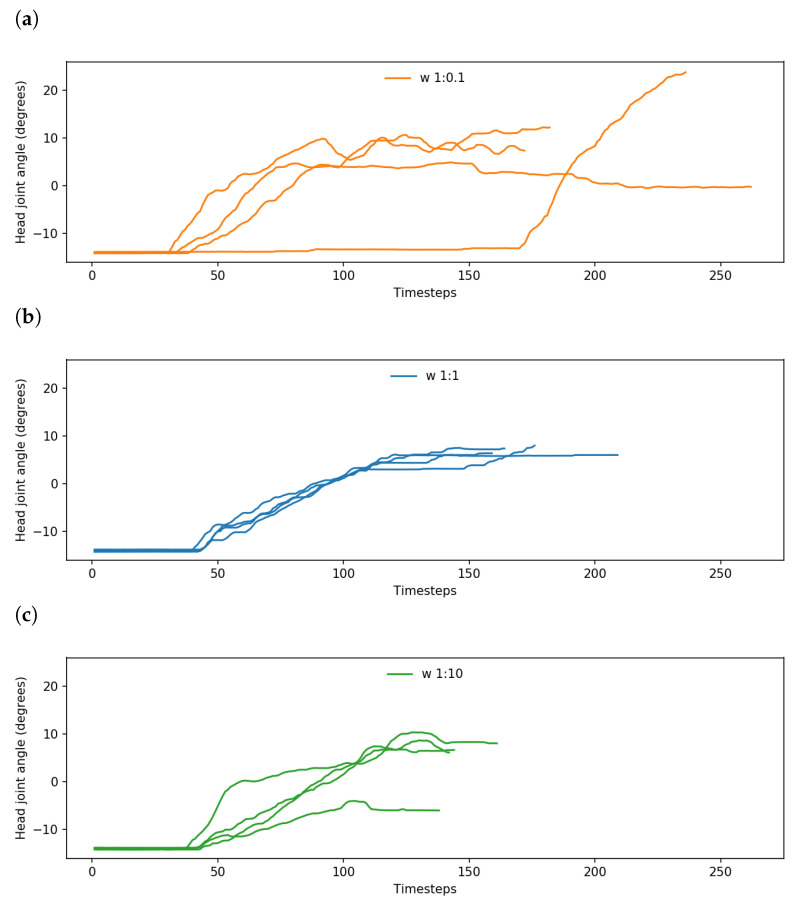
Recorded trajectories of the robot head joint angle as it tracks the object while executing the goal-directed plan. Each line represents one network. (**b**) Networks with *w* ratio 1:1, (**a**) networks with *w* ratio 1:0.1, and (**c**) networks with *w* ratio 1:10.

An example of this is shown in Figure 8a,b, which plot trajectories of one of the robot’s head joints while controlled by the 1:0.1 and 1:1 networks, respectively, for the same object and goal positions. As the robot’s head is constantly tracking the object independently of the network, while still being influenced by the rest of the robot’s movements, it is a good representation of the consistency and stability of the plan generation.

Networks with a *w* ratio of 1:1 output a tight bundle of trajectories, indicating learning of habituated motions, while the end point of each trajectory is also approximately the same with some variation in the length of trajectories. On the other hand, networks with a *w* ratio of 1:0.1 showed noticeably more variation in the output joint trajectory, as well as the end point and length of the trajectory, despite undertaking the same task with the same positions as the 1:1 networks.

Comparing networks with *w* ratios of 1:1 and 1:10, while the task performance is similar, the 1:1 networks tend to slightly outperform the 1:10 networks. We observed that this occurred largely because the 1:10 networks occasionally ignored sensorimotor observations and preferred goals. An example of this is shown in Figure 8c, which demonstrates that under the same conditions as the 1:1 networks, while 1:10 networks generated trajectories that were consistently shorter and stable, there were notable deviations from the tight bundle of trajectories shown by the 1:1 networks. In these cases, the robot either pushed the object to a preconceived grasping point, or moved the object to a completely different goal position from the preferred goal.

Finally, even in the best case, some goal displacement is measured. Apart from normal fluctuations in the stochastic network, the majority of this displacement comes from the scale of $\hat{g}$ not corresponding exactly to the goal area in the camera image. As can be seen in the image sequence in Figure 6, even though the network controlled the robot to place the object in line with its predicted exteroception, that position did not line up with $\hat{g}$. This offset was consistent, and did not influence subsequent results.

From the aforementioned results, it was surmised that the 1:1 networks, i.e., w=1 for both layers, presented the best task performance in the basic task. These networks were carried forward to the subsequent experiments. We also examined how the latent states represent variation in task trajectories with regard to different initial object and goal positions in Appendix A.

### 4.2. Experiment 2: Task Interference

In the previous experiment, we tested the robot with the same task as in training, with different object and goal positions to establish sufficient generalization and stability. However, in this experiment, we introduced a change in the robot’s task by means of human interference. The experimenter either moved the object just before the robot grasped it (task A) or the preferred goal $\hat{g}$ was changed after the robot placed the object at the previously specified goal position (task B). Subsequently, we refer to these tasks collectively as interference tasks, and these tasks were not present in the initial learning dataset. In this context, the task was independent of the objective of the robot, which always remained the same: to move the object to the goal position. In this study, the task undertaken by the robot comprised typical actions that the robot had to take in order to accomplish the goal. The task of picking up the object and placing it at the goal position, even if the positions change, are fundamentally the same set of actions, i.e., the same task. By requiring the robot to take a different series of actions, we changed the task.

In this experiment, initial conditions (object and goal positions) of each trial were identical to the previous experiment, and the four trained networks with *w* ratio 1:1 were used. In order to evaluate the degree of adaptation that is possible by PV-RNN without incremental learning, we modified the *w* values during plan generation. Table 2 shows the *w* values used in this experiment. In this table and subsequently, $w_{p}$ refers to the *w* value used in the past window, $w_{f}$ refers to the *w* value used in the future window, and $w_{t}$ refers to the *w* value used during training, i.e., $w_{t}=1.0$. The effect of this was expected to be similar to that of *w* in the the previous experiment, with $w_{p}$ affecting adaptation to sensorimotor observations, while $w_{f}$ should affect how strongly the preferred goal influences generation, compared to the learned prior. Each of the trained networks was tested in each *w* configuration on the five test cases, with the two aforementioned interference tasks, for a total of 100 trials per task.

#### 4.2.1. Experiment 2A: Object Moved before Grasp

In this experiment, at a fixed time step t=15 an audio cue signaled the experimenter to move the object to a predefined position. The objective of moving the object at this time step was to have the object position change suddenly, immediately before the object should have been grasped, thereby necessitating corrective action. This predefined position was different in each case. The goal position $\hat{g}$ remained unchanged. It was expected that the robot would experience a rapid change in exteroception, leading to increased evidence free energy. As the plan was updated, the free energy should decrease. The difference between this new task and the basic task with no interference was relatively small. It was hypothesized that with a lower meta-prior, the network may be able to better adapt its approximate posterior to match its sensorimotor observations, even it if deviated from its learned prior.

**Figure 9:**
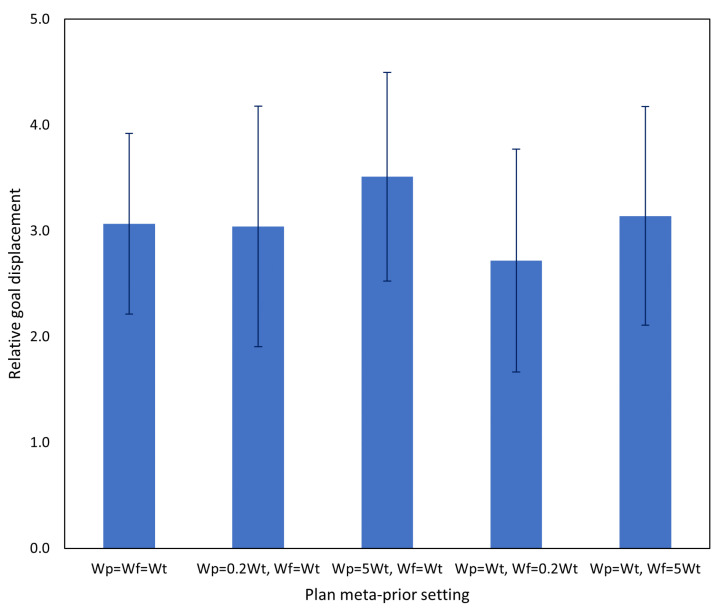
Relative goal displacement for five combinations of meta-priors in Table 2 for the robot undertaking interference task A. Relative goal displacement is relative to the same test cases without interference.

The result is shown in Figure 9. First, despite the relatively small change introduced by task A, overall task performance dropped significantly, with a goal displacement of approximately 300% of the baseline case. Second, changing the meta-prior setting in either the past or future window did not have a significant impact on task performance. Although the case in which the future meta-prior was reduced resulted in slightly better task performance, and cases in which the meta-prior was increased performed worse, the effect size was too small to be conclusive.

**Figure 10:**
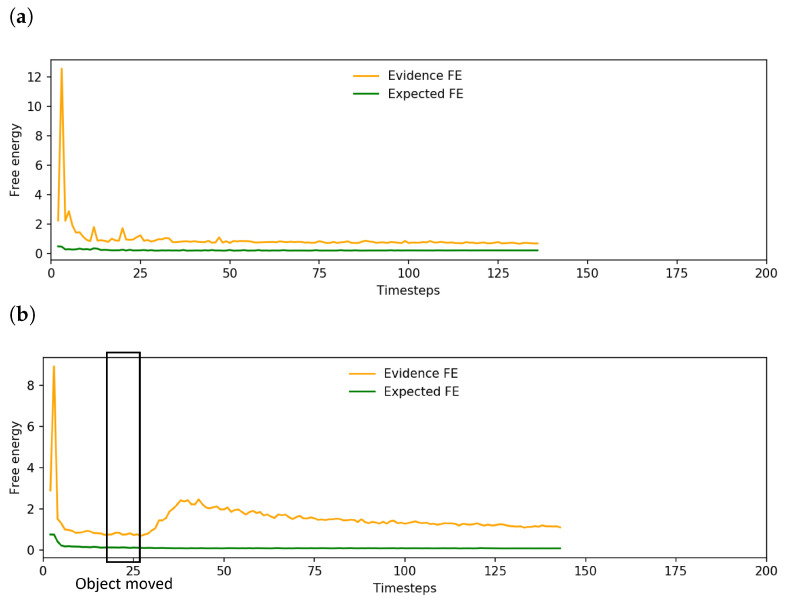
Examples of change in evidence free energy (orange line) and expected free energy (green line) during online plan generation. In each case, free energy was high until the network situated itself with the sensorimotor observations. (**a**) During a basic task (no interference). (**b**) During task A, with the period of time the object was moved by the experimenter marked in a black box; note the delay and slow rise in evidence FE due to the time taken for the exteroception to catch up to the new object position.

To investigate further, we examined the change in free energy of the networks undertaking the basic task, task A and task B. The basic case in Figure 10a shows the expected pattern of initially high free energy until the network situates itself, followed by a rapid drop to a low level of free energy. However, considering the free energy plot of a network undertaking task A (Figure 10b), there are two points that contributed to the reduced task performance.

First, we observed a noticeable delay from moving the object until the plan was updated. This was reflected as a lag in the increase in evidence free energy. Although the signal to move the object was given at t=15, human reaction time and the time to move the object to the new position added approximately 10 sensorimotor time steps until the object was in the new position. There was also additional delay from the object tracker controlling the robot head which moves to follow the object. As the object was moved just before the robot expected to grasp it, the robot occasionally pushed the object inadvertently during the delay which, in turn, further altered the exteroception.

**Figure 11:**
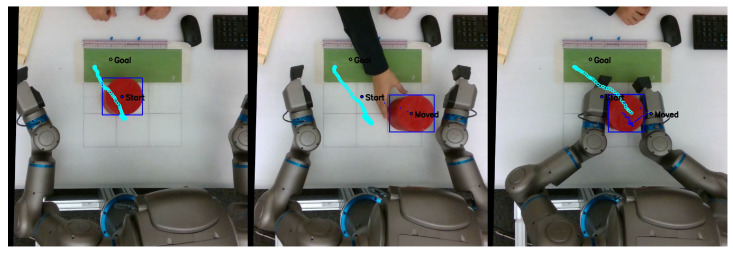
A sequence of three images showing an example of the robot undertaking the interference task A. Note that the predicted exteroception in cyan does not update immediately once the object is moved. The result is that the robot inadvertently pushed the object and grasped it incorrectly (with the edges of the end effector instead of at the center).

Following this delay in recognition of the new object position, the robot sometimes inadvertently grasped the object incorrectly, often using the edge of its end effectors instead of grasping the object firmly in the center. This problem was exacerbated in cases in which the robot was already touching the object before the plan could be updated. This caused the robot to inadvertently drop or misplace the object. An example of these issues can be seen in the image sequence in Figure 11. Thus, despite the trained networks being able to adapt to untrained object positions at the onset of planning, task performance is significantly impacted if the object is suddenly moved just before the robot reaches the expected object position.

#### 4.2.2. Experiment 2B: Goal Moved after Object Placed

In this experiment, at a fixed time step t=140 the goal position $\hat{g}$ was changed programmatically. The objective of changing the goal at this time step was that the object should already have been placed at the original goal, which would necessitate additional actions to move the object to the new goal. This task is significantly different than the basic task, as it requires grasping and placing the object a second time, making the trajectory significantly longer. As such, each trial was allowed to continue past the predicted distal step in order to allow time for the network to try to adapt its predictive posterior and generate a new plan.

**Figure 12:**
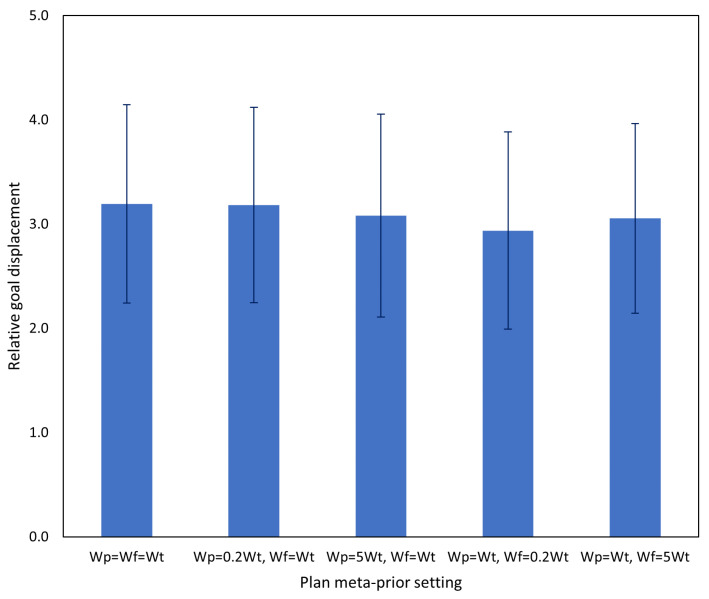
Relative goal displacement for five combinations of meta-priors in Table 2 for the robot undertaking interference task B. Goal displacement is relative to the same test cases without interference.

The result is shown in Figure 12. Results of this experiment are broadly similar to those of Experiment 2A, although with a very slight performance gain from reducing $w_{f}$.

**Figure 13:**
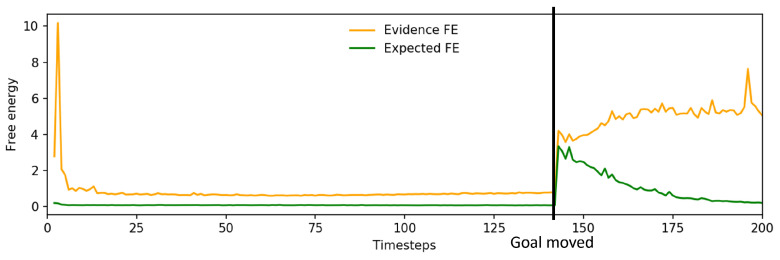
Example of change in evidence free energy (orange line) and expected free energy (green line) during online plan generation during task B. The time the goal position is changed marked by a black line. Note the sharp rise in both evidence and expected FE and the lack of drop in evidence FE.

In a few instances, the robot did spontaneously drag the object to the new goal position; however, this was not reliable. An example of this is shown in Figure 13. After the spike of expected free energy following the change in $\hat{g}$, it began to decline; however, evidence free energy continued to rise. This suggests that sensorimotor observations were being ignored, which tends to cause unstable future behavior. Alternatively, expected free energy would remain high and the robot would not react to the new goal position. In either case, it is apparent that the network was not able to generate action plans that minimize the error in both past observations and future goals.

From the preceding two experiments, it can be surmised that despite adequate performance on the basic task without human interference, when the situation changed due to human interference, the network by itself did not have sufficient adaptive capacity to adequately perform the new task on the fly. As such, in the following experiments, in which we introduced our incremental learning scheme, we used a single meta-prior configuration $w_{p}=w_{f}=w_{t}=1.0$.

### 4.3. Experiment 3: Incremental Learning

Finally, after evaluating the baseline performance of the model in the basic and interference tasks, we proceeded to incremental learning. First, it was necessary to collect tutoring examples of the new tasks. To accomplish this, the procedure from Experiment 2 was used with one of the trained networks as a representative network. Following this, three examples of task A were executed. For these tutoring examples, the object and goal positions were different from the test cases. The difference between this experiment and the previous experiment was that the human experimenter intervened and physically moved the robot in the following cases:

1. The robot attempted to grasp the object incorrectly;
2. The robot released the object before reaching the goal position;
3. The robot failed to respond to a change in goal position.

**Figure 14:**
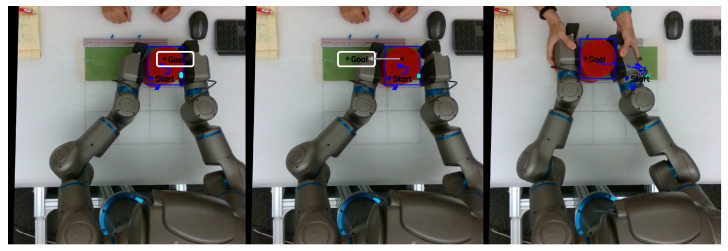
Example of an experimenter intervening to guide the robot when the goal position (highlighted in white) changes. When the robot did not respond to the new goal position, the experimenter pushed the robot’s end effectors in order to pick up the object, then pulled the robot’s arms toward the correct goal position before releasing the robot.

In the case of intervention, the human experimenter grasped the robot’s end effectors and exerted sufficient force to manipulate the robot into completing the task. Once tutoring began, the predicted distal step was ignored. At the end of each tutoring session, the robot saved the proprioceptive–exteroceptive sequence for incremental learning. This process was repeated for task B. An example of the experimenter conducting tutoring with the robot is shown in Figure 14.

Once a total of six tutoring examples were collected, the incremental learning process was started. This process was conducted sequentially, that is, task A was incrementally learned first, followed by task B. While the network parameters remain unchanged from the initial settings in Table 1, there were several alterations from the initial learning undertaken in Experiment 1. First, as noted in Section 3.4, in order to balance efficient learning of new tasks and retention of old tasks, the network used a process of mental rehearsal by generating sequences from its learned prior, analogous to the process undertaken in [41,54]. For the first incremental learning session, 57 rehearsal samples were generated and an additional 100,000 epochs of training were conducted for each network. For the second incremental learning session, based on the amount of training already completed and the fact that task B deviated further from the basic task and task A, the number of rehearsal samples generated using the networks after incrementally learning task A was reduced to 17. In preliminary studies, we found that reducing the number of rehearsal samples to increase the ratio of new tutoring samples was effective in efficiently learning longer tasks with significantly changed actions. With a smaller number of learning samples, the number of additional training epochs was also reduced to 75,000. The previous session’s training data were not used for subsequent incremental learning sessions. In addition, while network parameters were passed on between incremental learning sessions, the approximate posterior distributions were reset each time, along with the Adam optimizer momentum.

Finally, as in initial training, we undertook the following procedure to filter out poorly trained networks that might have been significantly overfitted or underfitted, as such networks can cause unsafe robot operation.

1. For each of the four trained networks, incremental learning of task A was conducted three times independently;
2. The resulting 12 networks were sorted by the loss at the end of training;
3. The top four and bottom four networks were discarded, leaving four networks to test;
4. Steps 1 to 3 were repeated for task B.

#### 4.3.1. Experiment 3A: Catastrophic Forgetting

A major potential issue with incremental learning is the phenomenon of catastrophic forgetting, in which the performance of previously learned tasks drops after incremental learning is conducted on a new task. In this case, both task A and the basic task could be affected by catastrophic forgetting. After following the aforementioned incremental learning procedure, we first evaluated the networks after the first incremental learning session (task A), and after the second incremental learning session (task A followed by task B) for catastrophic forgetting.

**Figure 15:**
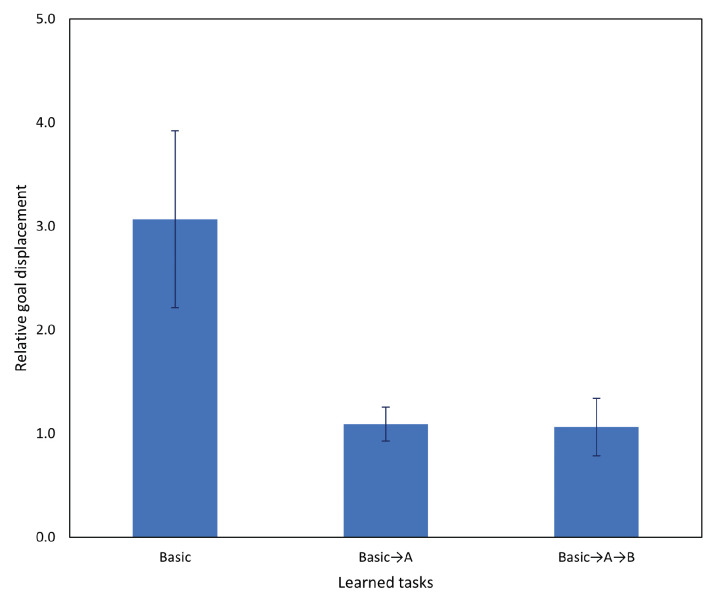
Relative goal displacement on task A using networks before incremental learning (Basic), after incremental learning of task A (Basic→A), and after subsequent incremental learning of task B (Basic→A→B).

Figure 15 shows the results of testing task A with the networks prior to incremental learning, the networks after the first incremental learning session, and the networks after the second incremental learning session. After incremental learning, task performance of the networks on task A returned to baseline levels (i.e., the equivalent task without interference). Additionally, while it was expected that performance on task A should be significantly improved immediately after incremental learning on tutoring examples of task A, the aforementioned results show that even after incrementally learning another task, task A performance was not significantly impacted. However, it is possible that adjusting of the number of rehearsal samples for each task may cause overfitting and may not be suitable in all cases. An alternative approach would be to increase the number of human tutoring samples until the desired behavior is exhibited, which may involve more rounds of incremental learning.

**Figure 16:**
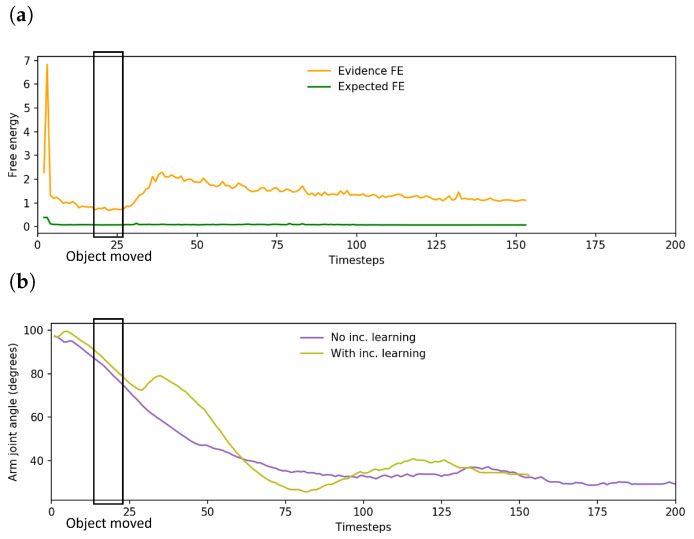
(**a**) Example of change in evidence free energy (orange line) and expected free energy (green line) during online plan generation, while the robot completes task A (object moved), after incremental learning. (**b**) Comparison of generated trajectories of an arm joint (right arm elbow) from a network prior to incremental learning (purple line) and after incremental learning (green line). The black box highlights when the object was moved. Note the sudden change in direction of the arm joint after the object moved, followed by a significantly changed trajectory compared to the output from the network without incremental learning, indicating repositioning of the end effector to compensate for the new object position.

Figure 16a shows a plot of the change in free energy while the robot undertook task A. While the change in free energy appeared very similar to the pattern observed prior to incremental learning as shown in Figure 10b, the updated plan that was generated as the evidence free energy was minimized resulted in far more accurate grasping of the object, leading to improved task performance. An example trajectory is shown in Figure 16b, which plots the trajectory of a single arm joint while the robot is controlled by a network before and after incremental learning. Visible changes in arm joint angle indicate that the network, after the previously discussed delay, generated a planned motion to reposition the end effector to correctly grasp the object at the new position.

**Figure 17:**
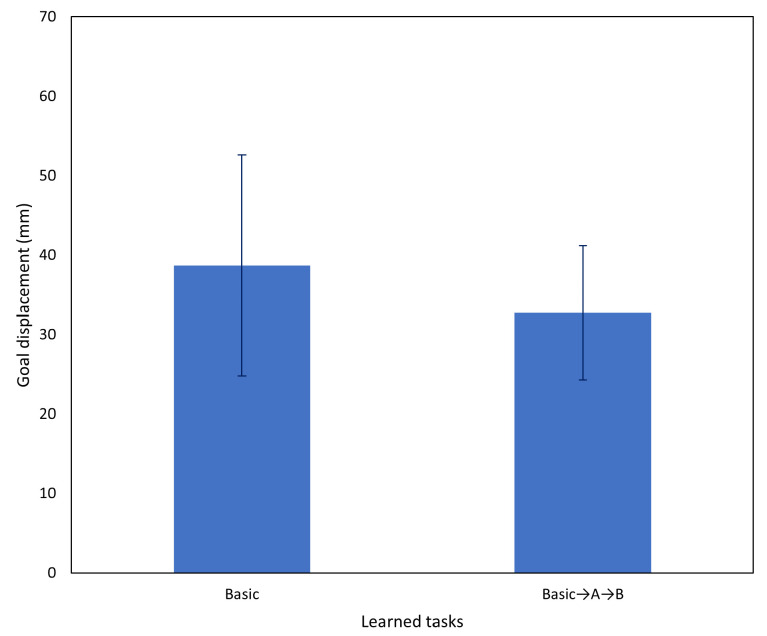
Goal displacement on the basic task using networks before incremental learning, and after incremental learning of task A followed by task B.

Figure 17 shows the results of testing on the basic (no interference) task using the networks after incremental learning compared to the networks before incremental learning. After two rounds of incremental learning, performance on the basic task did not degrade, and actually improved slightly. Based on the aforementioned results, subsequent testing was conducted using only the network after both incremental learning sessions.

#### 4.3.2. Experiment 3B: New Task Acquisition

**Figure 18:**
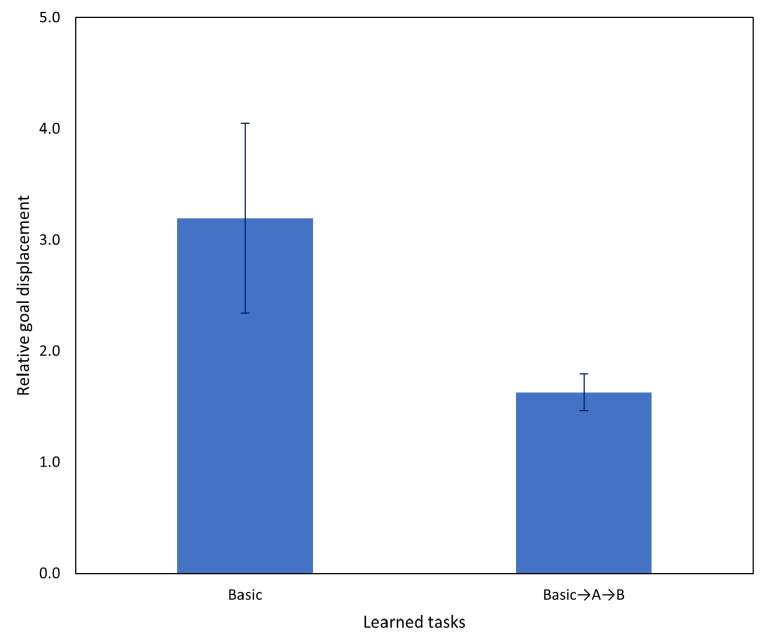
Relative goal displacement on task B using networks before incremental learning, and after incremental learning of task A followed by task B.

Following the significant improvement in task A after incremental learning in Experiment 3A, in this experiment, we evaluated the network on task B. Results comparing the network before and after incremental learning are shown in Figure 18 and, as in the previous experiment, we observed a significant task performance improvement after incremental learning.

**Figure 19:**
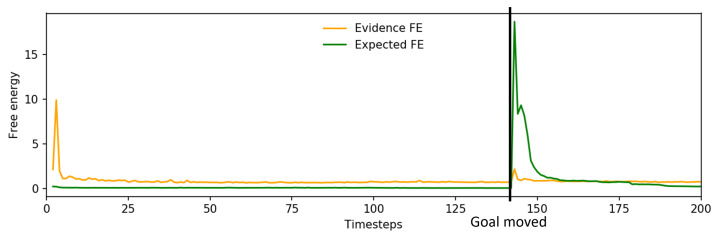
Example of change in evidence free energy (orange line) and expected free energy (green line) during online plan generation, while the robot completes task B (goal moved), after incremental learning.

Examining the changes in free energy during online plan generation (Figure 19), while the spike and drop in expected free energy is similar to that previously observed in Figure 13, the evidence free energy in this case also dropped back to levels close to those prior to interference in goal position, unlike in the case prior to incremental learning. This suggests that the network was able to adapt its future plan without ignoring sensorimotor observations, enabling generation of a more stable future trajectory for the robot and resulting in improved task performance.

Another matter to consider, particularly in our current study where a human is supervising and physically tutoring the robot, is the amount of human intervention required to ensure adequate task performance on more challenging tasks. After incremental learning, the amount of human intervention required should be reduced, consistent with improved task performance overall. Conditions for intervention by the experimenter are identical to those listed in Section 4.3.

**Figure 20:**
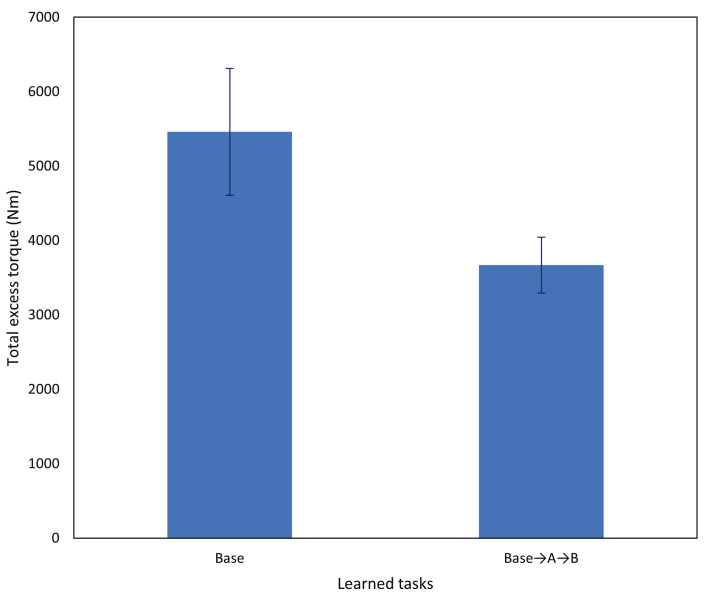
Total excess torque recorded by the robot during testing on task B using networks before incremental learning, and after incremental learning.

In this experiment, we considered the excess torque recorded by the robot while undertaking the task. The task undertaken is task B (goal position change) (Figure 20). Excess torque in this context refers to the difference in torque applied by the robot’s motors compared to the amount predicted by the robot controller’s internal dynamics model D, and is computed as $\mathrm{total}\;\mathrm{excess}\;\mathrm{torque}=\sum_{t}\sum_{j}\left|D\left(j,t\right)-{\mathrm{torque}}_{j,t}\right|$, where *j* is the robot joint index and *t* is the current time.

The robot controller consists of multiple levels, with the highest level receiving the generated predicted proprioception as a series of joint angles, which are subsequently converted into required torque levels after computing the appropriate acceleration and velocity to reach the commanded position from the current position. The dynamics model D is a fixed model that estimates the torque level that should be required by each joint motor in its current state, compensating for the force of gravity and internal friction. As the robot controller continually monitors joint states, when a deviation is detected, the commanded torque level can be adjusted to maintain the current state within a control loop. This commanded torque is converted by a low level controller into the current required to move each motor.

**Figure 21:**
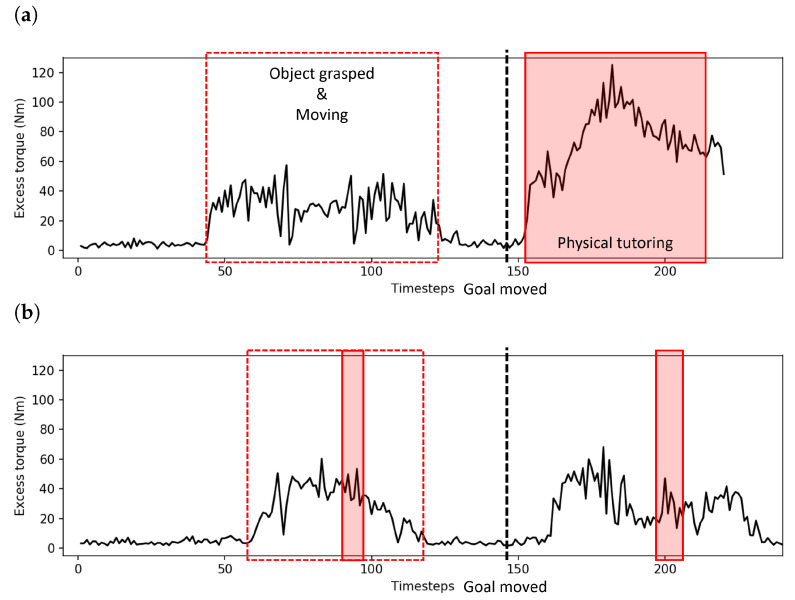
(**a**) Example of excess torque recorded over time, with the robot controlled by a network before incremental learning. Red dashed areas represent periods of time when the robot had grasped the object and was moving it. The dashed black line indicates when the goal position was changed. Red-shaded areas indicate when the experimenter was applying force in order to guide the robot to the goal. (**b**) Comparison of excess torque recorded over time, with the robot controlled by a network after incremental learning. The marked areas represent the same events as in (**a**).

Significantly lower excess torque was recorded by the robot, that is, less human intervention was required while undertaking the task. Some excess torque is always detected by the robot due to inaccuracies in the internal dynamics model and the force the robot itself exerts while grasping the object. Figure 21 shows an example of excess torque recorded by the arm joints, during task B. Time steps at which the robot was moving the object, the goal position change, and physical tutoring by the experimenter are annotated on the plots. Before incremental learning, a considerably longer time and larger magnitude of force was applied to the robot compared to the same situation after incremental learning. After incremental learning, a small amount of intervention was noted by the experimenter following the protocol described in Section 4.3; however, the small magnitude of the external force indicates that the intervention was not significant. Without experimental notes, it would be difficult to discern these interventions from noise from the sensors and fluctuations from normal interactions with the control loop.

From these results, we determined that after our incremental learning scheme was applied, not only did task performance significantly improve in newly acquired tasks, but task performance on previously learned tasks was retained. This was observed not only in a reduction in goal displacement to near baseline levels, but also in the reduced time and magnitude of human intervention required by the robot to reach the goal when testing on untrained positions. This suggests that subsequent human tutoring sessions should be easier or possibly unnecessary with additional parameter tuning.

## 5. Discussion

In our previous study [7], we discussed the possibility of investigating how a robot using our proposed model would respond to unexpected environmental changes, such as when the robot was unable to grasp the object due to external interference. The current study proposes a new scheme for a robot to conduct incremental learning of goal-directed actions utilizing our previously proposed model for online goal-directed action planning. Our proposed scheme addresses the two key issues of incremental learning as follows. First, it avoids the problem of catastrophic forgetting of previously learned tasks by means of mental rehearsal. This is accomplished by using the previously trained model to generate rehearsal samples. This approach also has the advantage of not requiring storage of old training data, which may not be practical in real-world applications. Second, it can efficiently learn new tasks from a small number of human tutoring examples by adjusting the number of rehearsal samples and tutoring samples, and by learning each new task sequentially. Tutoring samples consist of proprioception–exteroception sequences that are created when the robot appears to deviate from the task, and the human experimenter intervenes and physically guides the robot to complete the task. These objectives are accomplished using our T-GLean model and the PV-RNN network architecture, by first configuring network parameters to balance learning of stable habituated actions and generalization to different positions, and then adjusting the ratio of new tutoring samples and rehearsal samples to ensure new task acquisition in an efficient manner. Our testing showed that with a few tutoring examples, a relatively small number of rehearsal samples could be used to maintain the balance of new task acquisition and old task retention.

Our proposed learning scheme was evaluated in three phases. In the first experiment, we established baseline performance on a basic task of picking up an object and placing it at a specified goal position. This became the benchmark for task performance when interference was introduced. In two subsequent experiments, we evaluated the selected network configuration with different settings of the meta-prior on two new tasks that resulted when the human experimenter interfered with the robot’s environment while the robot was already executing a plan. The two tasks, designated A and B, corresponded to a relatively small change (the position of the object was changed just before the robot grasped it) and a larger change (the goal position was changed after the robot placed the object down) during the basic task. In the final set of experiments, we first conducted incremental learning following our proposed scheme by collecting a small number of examples of the human tutoring the robot to complete the new tasks. After tutoring samples were collected, incremental learning was performed sequentially, first on task A, and then on task B. Once incremental learning was completed, testing on tasks A and B was undertaken again, in addition to the original basic task without interference by the experimenter.

Results of these experiments established that even with networks that showed both stable generation of habituated actions and generalization to untrained positions, when faced with a sudden change in the environment, such as sudden movement of the object, task performance deteriorated significantly, as the network could not generate the necessary robot actions to compensate for conditions such as inadvertent contact with the object with the wrong part of the end effector. Even if the new object position could easily have been reached if the robot was reset to its home position and the network allowed to plan from scratch, the task with interference was sufficiently different from the original task in that it required new actions to perform adequately. After conducting incremental learning, on both interference tasks A and B, goal displacement returned to near the performance of the basic no-interference task. In addition, our results also showed that the networks, after incrementally learning A followed by B, retained both the original basic task and the previously learned task A. This confirms that following our proposed incremental learning scheme, by using a small number of human tutoring examples and mental rehearsal prior to each incremental learning phase, the model was able to incrementally learn new tasks without catastrophically forgetting previously learned tasks.

Apart from the significant improvement in task performance, evaluated by the difference in object position relative to the goal position at the end of the trial, we also analyzed the neural networks’ re-planning behavior following the rise and fall of evidence and expected free energy based on the interference received. While a drop in evidence free energy did not always correspond to an updated plan that adequately completed the task, a constantly elevated expected free energy or a rising evidence free energy indicated that the network was not able to reconcile its sensorimotor observations with the preferred goal and its learned prior. We generally observed a more stable decrease in both evidence and expected free energy after interference following incremental learning. Additionally, we quantified the amount of human intervention the robots required in order to complete the tasks in terms of the amount of force applied by the experimenter, and our results showed that significantly less human intervention was required for the robot to complete the task adequately after incremental learning. From this result, we concluded that as more incremental learning is undertaken, less human intervention is required for the robot to reach the goal in cases in which the robot encountered external interference.

Other studies have also considered the use of incremental learning in teaching robots in a human–robot collaborative setting, such as in [57], where the authors used Gaussian processes (GP) to determine the model’s task uncertainty in a manner similar to our use of free energy, although GPs were not used for model optimization. An intriguing proposed method [58] takes an active inference approach to reinforcement learning (RL) by imitation, using free energy instead of typical reward functions. Our proposed scheme utilizes human tutoring combined with mental rehearsal [14,16] for incremental learning, and integrates them with our active inference-based model that employs free-energy minimization in the latent space to find an optimal posterior predictive distribution. This scheme utilizing PV-RNN and error regression not only allows for efficient online plan generation, but also governs regularization in both the initial and incremental learning contexts, analogous to regularization approaches as in [41].

In the field of reinforcement learning, there is a significant body of work about goal-directed action planning. A well known example of this is Dreamer [31], a model-based RL agent that can learn a world model and then generate long-horizon behaviors using imagination in the latent space, similar to our approach of mental simulation. Dreamer has been developed further as DreamerV2 [59], which uses a discrete latent representation and KL-balancing to control the learning between prior and posterior, and DreamerV3 [60], which refined DreamerV2 and showed that with an optimized configuration, it could outperform specialized models on the same tasks. While Dreamer also applies variational free energy as its loss function, Dreamer’s goal-directed behavior is driven by reward prediction, unlike our approach, which can arbitrarily specify goal states and generate goal-directed actions by minimizing expected free energy. Other studies have also shown that an RL agent can learn a model of both the world and goals that can be accomplished [61], as well as multiple sub-goals that can be represented in latent space [33].

So-called incremental RL has attracted some attention, such as lifelong incremental reinforcement learning [62], a proposed method that encodes all experienced environment models in a latent space representation that can then be searched from the agent’s observations of the environment. Similarly, transfer learning of the RL agent’s environment model to new tasks, i.e., new reward functions, not only enables the agent to quickly learn new tasks with performance comparable to conventional Q-Learning and DQN [63], but also to improve performance in previously learned tasks, an analogous phenomenon that we have observed in this study.

Since incremental learning is a broad field of research, an interesting extension of the current work would be to evaluate other approaches for incremental learning. In this study, we adopted a bio-inspired approach based on mental rehearsal as a way of acquiring and retaining motor skills, integrating it with our active inference-based method of action planning and tutoring, as well as leveraging the PV-RNN architecture for regularization. While this approach was a logical extension of our previous work, it would be worth considering alternative approaches and methods with regard to incremental learning of new tasks, as well as further evaluation on a broader set of tasks and parameters. The key limiting factor, due to the nature of physical robot experiments, is the time required to gather necessary data with limited hardware resources. To this end, we may also consider utilizing some of the aforementioned reinforcement learning approaches, although these carry their own risks to robot hardware due to the need for a large number of trials. To alleviate this issue, we are considering a task utilizing a simulated version of our robot using interactions within the simulated space, which would make testing multiple different approaches tractable.

Continued future study could also consider other modalities such as the language modality, as suggested by Parr and Pezzulo [34]. In the current study, certain concepts such as understanding of constraints could only be inferred from activity within and actions generated by the neural network. By leveraging rich linguistic expressions, not only could more abstract and complex goals be represented, but the network could also make statements explicitly about its internal model of the world. Complex action structures involving compositionality, repeating patterns and variations in speed that are challenging to teach with a limited tutoring set could be represented. Finally, such a means of communication would give the robot a tool to tutor the human, making the adaptation of behaviors by both the human and the robot more concrete. Additionally, incorporating exteroception utilizing pixel-level data from the cameras could also enable more complex partially observable tasks.

## Figures and Tables

**Table 1:** PV-RNN parameters for used for all experiments. $R^{d}$ and $R^{z}$ refer to the number of deterministic (*d*) units and probabilistic (*z*) units, respectively. $w_{t=1}$ refers to the meta-prior setting at the first time step, which is set independently of the regular meta-prior *w*.

Layer *l*	$R^{d}$	$R^{z}$	τ	$w_{t=1}$
1	50	5	2	1.0
2	20	2	8	1.0

**Table 2:** Meta-prior *w* combinations tested in Experiment 2. $w_{p}$ is the *w* value used within the past window, and $w_{f}$ is the *w* value used in the future window. The setting string is the label each combination of $w_{p}$ and $w_{f}$ is given in subsequent results. Both PV-RNN layers use the same *w* values.

Setting	$w_{p}$	$w_{f}$
Wp = Wf = Wt	1.0	1.0
Wp = 0.2 Wt, Wf = Wt	0.2	1.0
Wp = 5 Wt, Wf = Wt	5.0	1.0
Wp = Wt, Wf = 0.2 Wt	1.0	0.2
Wp = Wt, Wf =5 Wt	1.0	5.0

**Figure A1:**
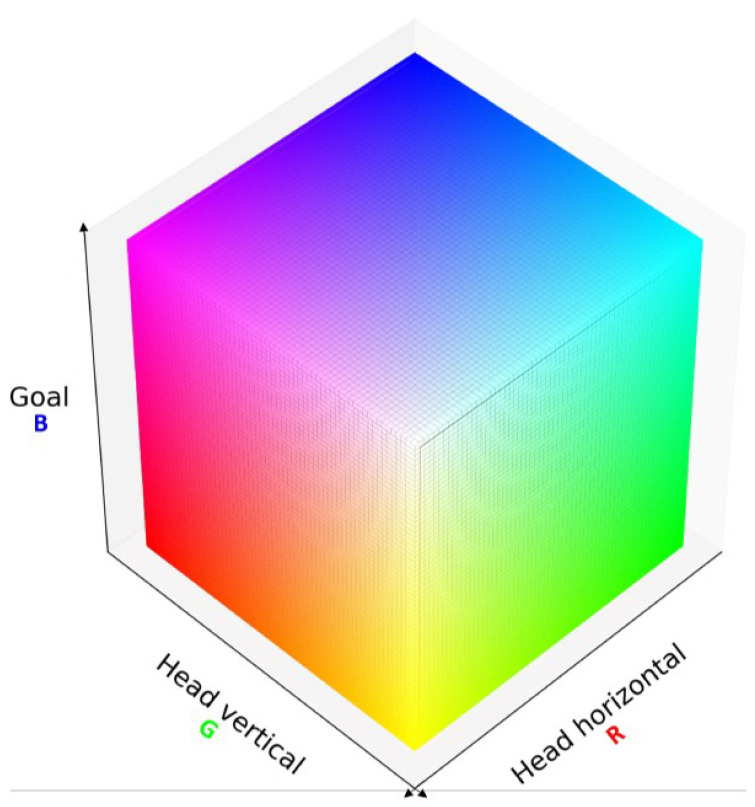
Color space representing possible combinations of 2D object position (derived from head angles) and 1D goal position.

**Figure A2:**
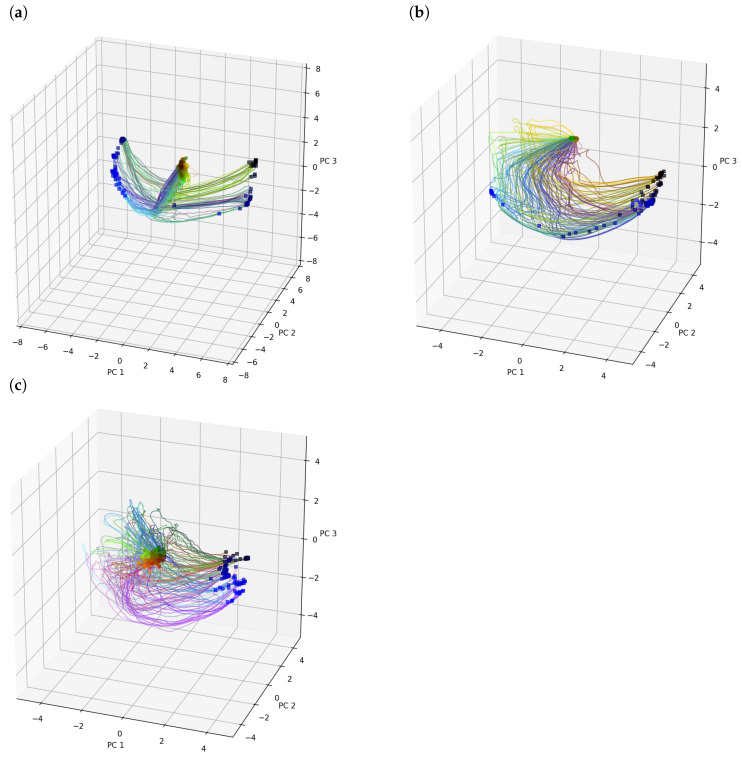
One hundred and twenty trajectories in latent space generated using trained prior distributions from Experiment 1, (**a**) 1:0.1 network, (**b**) 1:10 network, (**c**) 1:1 network.

## Data Availability

Instructions and source code for our model, along with the datasets used in this study, are available at https://github.com/oist-cnru/T-GLean-Inc (accessed on 3 September 2023).

## Appendix A

In this study, we examined the ability of the network to generalize to untrained object and goal positions, focusing on the task performance of the robot under several network configurations, with the implication that good generalization performance implies a robust representation in latent space. Under the concept of disentangled representation learning (DRL), it is expected that factors of variation are extracted from observations of the environment and then encoded in independent latent variables, and this may be important in achieving human-like generalization [64].

As shown in this Appendix, we examined latent spaces of three representative networks from Experiment 1, one from each of the meta-prior (*w*) ratios. With these networks, we undertook prior generation as shown in Figure 4 to generate 120 trajectories each, and conducted principal component analysis (PCA) on the deterministic latent variables (*d*) for the bottom layer at each timestep. As can be seen in Figure 2, the bottom layer *d* variables takes as input, not only the previous hidden state and current stochastic variables *z*, but also the top layer *d* variables. Factors of variation under consideration were the 2D head angles at the initial step t=1, which represents the anticipated initial object position as also demonstrated in Figure 8, and the 1D anticipated goal position at the distal step.

In the following figures, we represent the trajectories using a red–green–blue (RGB) color representation. Each color channel is mapped to one of the aforementioned factors of variation, with values normalized. The resulting space of possible position combinations was then represented as a cube of smoothly varying colors, an example of which is shown in Figure A1. In this representation, it follows that similar colors reflect similar trajectories.

Each trajectory was plotted in latent space with the first three principal components. There are three components to the trajectory:

1. The beginning of the trajectory is represented by a circle marker (•) using the initial object position colors (RG = 2D object position, B = 0);
2. The end of the trajectory at the distal step represented by a square marker (■) and using the goal position color (RG = 0, B = goal position);
3. The trajectory itself is identified using all color channels (RG = 2D object position, B = goal position).

Figure A2c shows trajectories in latent space for a *w* ratio 1:1 network. The start of the trajectories are visible in a cluster in the center, where there is an even distribution of starting points in both space and color. From the initial step, the trajectories spread out and curve toward the distal step cluster on the right. Although there is some variability in the trajectories, the start and end points show the expected color shifting, suggesting good disentanglement of the three factors of variation with respect to the three principal components of the latent space.

Compared to the *w* ratio 1:1 network, the *w* ratio 1:0.1 network in Figure A2a shows a planar structure with trajectories splitting into two distinct clusters of goal positions. The color distribution of trajectories is also noticeably skewed, lacking in reds compared to the *w* ratio 1:1 network in Figure A2c, suggesting an inadequate learning of the task distribution. This may explain the poor task performance exhibited by this network configuration.

Finally, Figure A2b shows the latent space trajectories for the network with *w* ratio 1:10 and, despite having task performance close to the 1:1 network, it shows a latent structure with similarities to the 1:0.1 network. Trajectories with different goal positions terminate on a wide arc; thus, based on the goal position, the trajectory in latent space may be quite different. The color distribution also appears to be slightly skewed, compared to Figure A2c.

While in this study we did not focus on the internal dynamics of the network, our inspection of the networks under different meta-prior settings showed significant differences in latent space representations that may correlate with the observed behavior of the robot. In future work, we would like to further examine how internal dynamics develop during different phases of learning.
